# Elevated methylation of the vault RNA2-1 promoter in maternal blood is associated with preterm birth

**DOI:** 10.1186/s12864-021-07865-y

**Published:** 2021-07-10

**Authors:** Young-Ah You, Eun Jin Kwon, Han-Sung Hwang, Suk-Joo Choi, Sae Kyung Choi, Young Ju Kim

**Affiliations:** 1 Department of Obstetrics and Gynecology, Ewha Medical Research Institute, Ewha Womans University Medical School, 07985 Seoul, Korea; 2 Graduate Program in System Health Science and Engineering, Ewha Womans University, 03760 Seoul, Korea; 3 Department of Obstetrics and Gynecology, Research Institute of Medical Science, Konkuk University School of Medicine, 143-729 Seoul, Korea; 4 Department of Obstetrics and Gynecology, Samsung Medical Center, Sungkyunkwan University School of Medicine, 135-710 Seoul, Korea; 5 College of Medicine, The Catholic University of Korea, 505 Banpo-dong, Seocho-gu, 137-040 Seoul, Korea; 6 Department of Obstetrics and Gynecology, Ewha Womans University Mok Dong Hospital, 158-051 Seoul, South Korea

**Keywords:** DNA methylation, Maternal blood, VTRNA2-1, Preterm birth, miR-886

## Abstract

**Background:**

Preterm birth, defined as parturition before 37 completed weeks of gestation, is associated with an increased risk of neonatal complications and death, as well as poor health and disease later in life. Epigenetics could contribute to the mechanism underlying preterm birth.

**Results:**

Genome-wide DNA methylation analysis of whole blood cells from 10 women (5 term and 5 preterm deliveries) was performed using an Illumina Infinium HumanMethylation450 BeadChips array. We identified 1,581 differentially methylated CpG sites in promoter regions between term and preterm birth. Although the differences were not significant after correcting for multiple tests, seven CpGs on the genomically imprinted vault RNA2-1 (VTRNA2-1; also known as non-coding RNA, nc886 or miR-886) showed the largest differences (range: 26–39 %). Pyrosequencing verification was performed with blood samples from pregnant women recruited additionally (39 term and 43 preterm deliveries). In total, 28 (34.1 %) samples showed hypomethylation of the VTRNA2-1 promoter (< 13 % methylation), while 54 (65.9 %) samples showed elevated methylation levels between 30 and 60 %. Elevated methylation of VTRNA2-1 promoter was associated with an increased risk of preterm birth after adjusting for maternal age, season of delivery, parity and white blood cell count. The mRNA expression of VTRNA2-1 was 0.51-fold lower in women with preterm deliveries (*n* = 20) compared with women with term deliveries (*n* = 20).

**Conclusions:**

VTRNA2-1 is a noncoding transcript to environmentally responsive epialleles. Our results suggest that elevated methylation of the VTRNA2-1 promoter may result in increased risk of PTB caused by the pro-inflammatory cytokines. Further studies are needed to confirm the association of VTRNA2-1 methylation with preterm birth in a large population, and to elucidate the underlying mechanism.

**Supplementary Information:**

The online version contains supplementary material available at 10.1186/s12864-021-07865-y.

## Background

Preterm birth (PTB) is defined as parturition before 37 weeks of gestation and approximately 15 million babies are born prematurely each year [1]. In Korea, the rate of PTB has continuously increased, from 4.8 % to 2008 to 7.8 % in 2018 [2, 3]. To date, clinicians and researchers have made great efforts to improve the identification of women at risk for PTB before its occurrence, as well as to develop therapeutics for its prevention. However, the early identification of PTB and therapy to mitigate its risk remain controversial [4, 5].

PTB is associated with an increased risk of complications and neonatal death, as well as poor health and disease later in life [6–8]. Spontaneous PTB has a range of contributing risk factors, including infection, undernutrition, stress, and substance use [9], which are linked by two common pathways [10]. First, inflammatory and neuroendocrine pathways are activated in response to stress or stress-related behaviours, such as smoking [11], causing the upregulation of inflammatory cytokine production [11]. Second, cytokine-prostaglandin cascades are activated in response to infection [10]. Due to the involvement of inflammatory mechanisms in both pathways, previous studies investigating potential biomarkers for PTB have focused on inflammatory mediators [12, 13].

Genome-wide DNA methylation analysis may provide information on the mechanism underlying PTB and represents a new approach for biomarker discovery [14–16]. DNA methylation in the blood may change according to conditions such as inflammation, and multiple studies have shown that differential DNA methylation is related to smoking status, as well as to obesity and various other diseases [17]. Moreover, the ability to assess the epigenome has resulted in the identification of epigenetic signatures of the intrauterine environment, which are affected by smoking, stress, nutrition, body mass index (BMI), and medication use over several years [18–22]. Many studies have suggested that differentially methylated genes are involved in the genetic and environmental contributions to PTB and chronic disease risk. DNA methylation changes in the amnion or foetal tissue were determined to be partially involved in the physiological process of PTB and in foetal development [23, 24]. Thus, the evaluation of genes containing these differentially methylated sites may be useful to identify biological pathways involved in PTB, thereby facilitating the identification of clinically informative biomarkers for the prediction of PTB. Accordingly, we investigated the association between blood DNA methylation and preterm birth among Korean women. We identified the seven CpG sites with a difference in VTRNA2-1 promoter methylation levels between preterm and term samples.

VTRNA2-1 (VTRNA2-1, also called nc886 or miR-886) is a 108-nucleotide noncoding transcript that is epigenetically controlled via 18 CpG sites of its promoter, and can exert either tumor suppression or oncogenic functions depending on cell types of cancers [25]. Intriguing aspects of the epigenetic regulation of this locus, include its dependence on the parental origin of the allele, and its sensitivity to the periconceptional environment [26, 27]. Here, we report that the elevated methylation of VTRNA2-1 promoter, validated by pyrosequencing after an epigenome-wide analysis, is associated with preterm birth.

## Results

### Methylation array in the peripheral blood of women delivering at term and preterm

A genome-wide analysis of DNA methylation was performed to search for candidate differentially methylated genes in maternal blood at the time of delivery. A total of 87,507 CpG sites were tested on the CpG island promoters (except SNP-affected CpG sites) and the results showed that 1,581 sites had significantly different levels of methylation (*p* < 0.05) between term and preterm groups (Table S1). Although we found no differential methylation (DM) of these CpGs after correcting for multiple testing, seven CpG sites in the VTRNA2-1 promoter showed the largest differences (26–39 %) between PTB and term samples (*p* < 0.05) (Table 1). In addition, these seven CpG sites showed elevated methylation (range: 25.0–55.1 %) in all 5 samples of term deliveries and 1 sample of preterm delivery, and hypomethylation (range: 2.6– 14.1 %) in 4 samples of preterm deliveries (Figure S1).

**Table 1:** The seven CpGs on VTRNA2-1 with the largest differences in methylation between term and preterm samples (*n* = 10)

Target ID†	CHR	Position	Functional location	Mean β value in term samples (*n* = 5)	Mean β value in preterm samples (*n* = 5)	Difference*	*p*-value^a^	*q*-value^b^
cg26328633	5	135,416,394	TSS200	0.125	0.520	0.395	0.014	0.050
cg25340688	5	135,416,398	TSS200	0.121	0.508	0.387	0.014	0.050
cg06536614	5	135,416,381	TSS200	0.129	0.492	0.363	0.020	0.050
cg00124993	5	135,416,412	TSS200	0.114	0.471	0.357	0.014	0.050
cg26896946	5	135,416,405	TSS200	0.192	0.503	0.311	0.013	0.050
cg04481923	5	135,416,205	1stExon;3’UTR	0.122	0.389	0.267	0.015	0.050
cg18678645	5	135,416,331	TSS200	0.115	0.376	0.261	0.051	0.050

Gene ID of VTRNA2-1 is 100,126,299

*CHR* Chromosome

^a^Mann–Whitney U-test, *p* < 0.05

^b^The false discovery rate (FDR) was controlled using the Benjamini–Hochberg correction (*q* < 0.05)

†Name of the probe

*Difference between mean β values of preterm births and term births

### Validation of VTRNA2-1 methylation level by pyrosequencing

Subsequently, we performed pyrosequencing to investigate the association between VTRNA2-1 methylation (cg04481923) and PTB in maternal blood samples (term, *n* = 39; preterm, *n* = 43). The primer sets for the VTRNA2-1 CpG sites were designed using PSQ Assay Design software (Biotage AB, Uppsala, Sweden). We performed pyrosequencing on cg04481923, where the primer set was designed with the highest score among seven CpGs. Table 2 shows the clinical characteristics of the 82 pregnant women. Maternal age ranged from 27 to 39 years in women with term deliveries, and from 22 to 43 years in women with PTB. Among the preterm delivery mothers, 21 were mothers with preterm premature rupture of membrane (pPROM), and 22 were mothers with preterm labor (PTL). The mean gestational age and BMI of women with term births were 39 weeks, 4 days and 26.0, respectively, compared to 29 weeks, 1 day and 23.8, respectively, in women with PTB. The incidence of C-section did not differ significantly between the term and preterm groups according to the chi-square test (*p* > 0.05). However, white blood cell (WBC) count and monocyte portion in the maternal blood were significantly different between term and preterm delivered women.

**Table 2:** Clinical characteristics of the study groups (*n* = 82)

	Term (≥ 37, *n* = 39)	Preterm (< 37, *n* = 43)	*p*-value
	Mean ± SD	Mean ± SD	
Maternal age	31.7 ± 2.8	30.9 ± 4.6	0.418
BMI at delivery	25.9 ± 3.3	24.1 ± 4.2	0.037^a^
Gravidity			0.471
0	16 (41.0)	15 (34.9)	
1	23 (59.0)	28 (65.1)	
Parity			0.118
Nulliparous	22 (56.4)	22 (51.2)	
Multiparous	17 (43.6)	21 (48.8)	
Delivery season			0.021*
Spring, *n* (%)	5 (12.8)	12 (27.9)	
Summer, *n* (%)	3 (7.7)	11 (25.6)	
Autumn, *n* (%)	19 (48.7)	13 (30.2)	
Winter, *n* (%)	12 (30.8)	7 (16.3)	
Mode of delivery			0.599
Vaginal, *n* (%)	24 (61.5)	24 (55.8)	
C-section, *n* (%)	15 (38.5)	19 (44.2)	
Education (*n* = 68)			0.096
Below high school	8 (20.5)	13 (44.8)	
College or more	31 (79.5)	16 (55.2)	
Gestational age	39.4 ± 1.0	29.1 ± 2.8	<0.001^a^
White blood cell, (x10^3^ cells/μL)	10.10 ± 3.00	12.21 ± 3.83	0.008 ^a^
Granulocyte, (%)	74.65 ± 7.44	77.81 ± 7.01	0.066
Lymphocyte, (%)	18.01 ± 5.78	21.55 ± 34.72	0.548
Monocyte, (%)	6.86 ± 2.01	5.81 ± 1.76	0.021 ^a^
Mycoplasma			0.702
Positive, *n* (%)	2 (5.4)	3 (8.6)	
Negative, *n* (%)	35 (94.6)	32 (91.4)	
Ureaplasma (*n* = 72)			<0.001*
Positive, *n* (%)	0 (0.0)	13 (37.1)	
Negative, *n* (%)	37 (100.0)	22 (62.9)	
Chorioamnionitis (*n* = 67)			<0.001*
Positive, *n* (%)	0 (5.1)	17 (56.7)	
Negative, *n* (%)	37 (100)	13 (43.3)	
Birth weight	3310.5 ± 394.7	1385.7 ± 504.1	<0.001^a^
Sex			0.654
Male, *n* (%)	20 (51.3)	24 (55.8)	
Female, *n* (%)	19 (48.7)	19 (44.2)	
Apgar 1 min	9.5 ± 0.8	5.9 ± 2.4	<0.001^a^
Apgar 5 min	9.9 ± 0.2	7.5 ± 2.1	<0.001^a^

Data are shown as the mean ± SD for continuous variables and as n (%) for categorical data

*BMI* body mass index

^a^Student’s t-test

*χ^2^ test

**Fig. 1:**
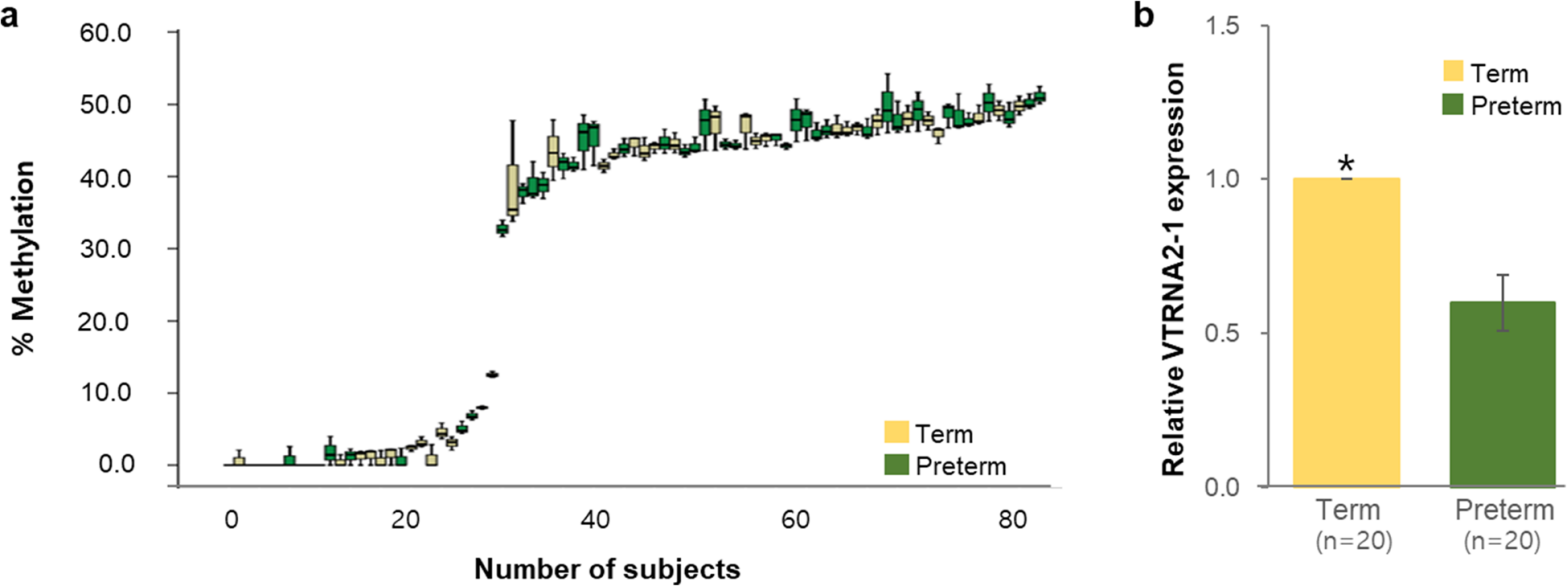
Methylation differences in VTRNA2-1 between term and preterm samples. **a** Rank plot of three methylated sites on VTRNA2-1 by bisulphite pyrosequencing. **b** Relative expression levels of VTRAN2-1 in maternal blood (Term, *n* = 20; Preterm, *n* = 20)

Table 3 shows the levels of DNA methylation of three CpG sites on VTRNA2-1 promoter following pyrosequencing; two CpG sites differed significantly between pregnant women with term births and PTBs (*p* < 0.05). These three CpG sites on VTRNA2-1, identified in all samples, were concordant with hypomethylation (range: 0–13 %, *n* = 28) or elevated methylation (range: 30–60 %, *n* = 54) (Fig. 1a). Blood samples from women who delivered preterm infants were more likely to exhibit elevated methylation (> 30 %) of VTRANA2-1 than women who delivered at term. In addition, the relative expression level of VTRNA2-1 was 0.51-fold lower (*p* < 0.05) in PTB women (*n* = 20) compared to those with term deliveries (*n* = 20) (Fig. 1b). The methylation levels of the VTRNA2-1 promoter were inversely correlated with levels of its expression (*r* = -0.741, *p* < 0.01).

**Table 3:** Comparison of specific DNA methylation sites between women with term and preterm births after pyrosequencing

Variable	Target ID† of differentially methylated site	Term (*n* = 39)	Preterm (*n* = 43)	*p*-value
VTRNA2-1_pos1	cg04481923	25.8 ± 20.9	33.9 ± 18.6	0.065
VTRNA2-1_pos2		26.3 ± 22.2	35.2 ± 19.5	0.045^a^
VTRNA2-1_pos3		27.4 ± 22.3	36.4 ± 19.8	0.026^a^

Data are presented as the mean ± SD

VTRNA2-1_pos1, _pos2, and _pos3 were analyzed by pyrosequencing as CpG sites of chromosome 5:135,416,205, chromosome 5:135,416,207, and chromosome 5:135,416,214, respectively

*VTRNA2-1* vault RNA 2 − 1

^a^Mann–Whitney U-test, *p* < 0.05

†Name of the probe

To evaluate the clinical relevance of VTRNA2-1 promoter methylation levels, we analysed its association with the demographic and clinical data of pregnant women. The elevated methylation of the VTRNA2-1 promoter was significantly associated with the diagnosis of pPROM, a main cause of PTB (Table 4).

**Table 4:** Correlation analysis of VTRNA2-1 methylation levels and risk factors of preterm birth

Variable	Maternal age	Education	Parity	Diagnosis^b^	WBC
VTRNA2-1_pos1	0.183	-0.107	-0.019	0.273^*^	0.105
VTRNA2-1_pos2	0.169	-0.095	-0.020	0.279^*^	0.104
VTRNA2-1_pos3	0.174	-0.147	-0.009	0.293^**^	0.094
Hypo vs. Elevated^a^	0.179	-0.128	-0.011	0.246^*^	0.075

The association between the categorical data for VTRNA2-1 methylation and the categorical data for preterm birth risks were analyzed using Fisher’s exact test. The association between the others and VTRNA2-1 methylation were analyzed using Pearson correlation analysis

VTRNA2-1_pos1, _pos2, and _pos3 were analyzed by pyrosequencing as CpG sites of chromosome 5:135,416,205, chromosome 5:135,416,207, and chromosome 5:135,416,214, respectively

^a^It divided two groups as hypomethylation (< 13 %) and elevated methylation (30–60 %)

^b^Diagnosis were analysed by substituting 1 for PTL, a nominal variable, and 2 for pPROM

^*^p < 0.05

^**^p < 0.01

### Preterm birth-related DNA methylation changes in maternal blood

Table 5 shows the association between DNA methylation sites and PTB, as determined by logistic regression analysis. It was divided into two groups based on the methylation level of VTRNA2-1 promoter: a hypomethylated group (< 13 %, *n* = 28) and an elevated methylated group (30–60 %, *n* = 54). In total, 28 (34.1 %) samples showed elevated methylation of the VTRNA2-1 promoter (< 13 % methylation by pyrosequencing), while 54 samples (65.9 %) showed a methylation level of 30–60 %. Based on these results, the patients were divided into hypomethylation and elevated methylation groups. The elevated methylation of VTRNA2-1 promoter was associated with a significantly increased risk of PTB compared with hypomethylation after adjusting for maternal age, season of delivery, parity and white blood cell count (adjusted OR = 3.358, 95 % CI 1.114–10.126). Interestingly, younger women in the VTRNA2-1 hypomethylation group were more likely to have preterm deliveries (*p* < 0.05, Table 6). Moreover, women with preterm deliveries in the elevated methylation group had lower BMIs and higher WBC counts and were also more likely to deliver during spring or summer than autumn or winter (*p* < 0.05).

**Table 5:** Logistic regression analysis of the association between cg04481923 methylation level in VTRNA2-1 gene and preterm births (*n* = 82)

Methylation	β-value	SE	Odds ratio	95 % CI	*p-*value
Age	-0.057	0.035	0.945	0.882–1.012	0.104
Season	-0.582	0.238	0.559	0.350–0.892	0.015
Parity	0.389	0.313	1.475	0.798–2.727	0.215
VTRNA2-1^a^	1.185	0.574	3.269	1.061–10.069	0.039
WBC count	0.211	0.075	1.235	1.066–1.431	0.005

β-values and SE estimated using the multivariable logistic regression model

*VTRNA2-1* vault RNA 2 − 1

^a^It was divided into two groups based on the methylation level of VTRNA2-1 promoter: a hypomethylated group (< 13 %, *n* = 28) and an elevated methylated group (30–60 %, *n* = 54)

**Table 6:** Clinical characteristics of VTRNA2-1 hypomethylation and elevated methylation groups (*n* = 82)

	Hypomethylation†			Elevated methylation‡		
	Term (*n* = 17)	Preterm (*n* = 11)	*p*-value	Term (*n* = 22)	Preterm (*n* = 32)	*p*-value
Maternal age	31.7 ± 2.7	28.2 ± 2.5	0.002	31.7 ± 3.1	31.9 ± 4.7	0.812
BMI	26.3 ± 3.3	24.5 ± 5.4	0.320	25.8 ± 3.3	23.5 ± 3.4	0.024
Gravidity*			0.954			0.412
0	6 (35.3)	4 (36.4)		10 (45.5)	11 (34.4)	
1	11 (64.7)	7 (63.6)		12 (54.5)	21 (65.6)	
Parity*			0.761			0.026
Nulliparous	9 (52.9)	5 (45.5)		13 (59.1)	17 (53.1)	
Multiparous	8 (47.1)	6 (54.5)		9 (40.9)	15 (46.9)	
Delivery season*			0.775			0.009
Spring, *n* (%)	4 (23.5)	4 (36.4)		1 (4.5)	8 (25.0)	
Summer, *n* (%)	1 ( 5.9)	0 (0.0)		2 (9.1)	11 (34.4)	
Autumn, *n* (%)	9 (52.9)	5 (45.5)		10 (45.5)	8 (25.0)	
Winter, *n* (%)	3 (17.6)	2 (18.2)		9 (40.9)	5 (15.6)	
Mode of delivery*			0.435			0.264
Vaginal, *n* (%)	9 (52.9)	8 (72.7)		15 (68.2)	16 (50.0)	
C-section, *n* (%)	8 (47.1)	3 (27.3)		7 (31.8)	16 (50.0)	
Education			0.417			0.226
Below high school	3 (17.6)	3 (37.5)		5 (22.7)	10 (47.6)	
Above college	14 (82.4)	5 (62.5)		17 (77.3)	11 (52.4)	
Gestational age	39.4 ± 1.0	29.3 ± 2.4	< 0.001	39.4 ± 1.4	29.1 ± 2.9	< 0.001
WBC (x10^3^)	10.3 ± 3.3	11.9 ± 3.0	0.218	9.9 ± 2.9	12.3 ± 4.0	0.028
Ureaplasma*			0.005			0.002
Negative	15 (88.2)	4 (36.4)		22 (100.0)	18 (56.3)	
Positive	0 (0.0)	5 (45.5)		0 (0.0)	8 (25.0)	
Birth outcome						
Body weight (kg)	3.5 ± 0.4	1.2 ± 0.3	< 0.001	3.2 ± 0.3	1.4 ± 0.5	< 0.001
Sex*			0.934			0.783
Male, *n* (%)	9 (52.9)	6 (54.5)		11 (40.0)	18 (56.3)	
Female, *n* (%)	8 (47.1)	5 (45.5)		11 (60.0)	14 (43.8)	
Apgar 1 min	9.3 ± 0.9	6.3 ± 1.8	< 0.001	9.7 ± 0.5	5.7 ± 2.5	< 0.001
Apgar 5 min	9.9 ± 0.3	7.8 ± 1.6	< 0.001	10.0 ± 0.0	7.4 ± 2.2	< 0.001

Data are presented as the mean ± SD for continuous variables and as n (%) for categorical variables

*BMI* body mass index

^a^Student’s t-test, *p* < 0.05

*χ^2^ test, *p* < 0.05

†Hypomethylation, < 13% methylation of VTRNA2-1

‡Elevated methylation, methylation level of 30–60%

## Discussion

We examined PTB-related DNA methylation changes through genome-wide methylation analysis of maternal blood. Our results showed for the first time that elevated methylation of the VTRNA2-1 promoter in maternal blood is more apparent in pPROM samples than PTL samples, and may increase risk of PTB. In concordance with methylation status, its expression was downregulated in preterm blood and upregulated in term blood samples. Our results suggest that elevated methylation of VTRNA2-1 promoter is susceptible to PTB.

PTB remains the leading cause of childhood morbidity and death. Its aetiology remains unclear; however, significant advances have been made in the identification of biomarkers to predict high-risk pregnancies resulting in PTB. One study using genome-wide methylation analysis reported that maternal methylation loci may serve as a biomarker for PTB, whereas cord blood methylation levels are not associated with PTB [28]. However, other studies have reported that numerous CpG sites in the cord blood are differentially methylated in relation to gestational age [29, 30]. Changes in DNA methylation caused by environmental factors regulate gene transcription and can play a role in a variety of diseases [31]. Studies have suggested that maternal factors, including socioeconomic status [11], pre-pregnancy BMI [32], smoking during pregnancy [33] and nutrition status [34] affect the risk of PTB. DNA methylation partially explains the effects many of these factors [32–34]. In this study, we found associations of maternal BMI, delivery season, and WBC count with PTB; however, these factors did not influence the DNA methylation levels of VTRNA2-1 in maternal blood.

The main pathways of initiation of PTB are fetal and maternal tissue activation by cervical insufficiency, stress, inflammation, and immune dysregulation. These risks result in myometrium contractions and/or rupture of the fetal membranes by the release of prostaglandins and interleukins (ILs) [35, 36]. In this study, elevated methylation of the VTRNA2-1 promoter was analysed in blood samples from 18 women out of 21 pregnant women diagnosis with pPROM, and 17 of 27 blood samples were analysed for elevated methylation in PTL. pPROM is the rupture of the membrane before the onset of labor before 37 weeks of gestation and accounts for one-third of all PTBs [37]. High levels of pro-inflammatory cytokines such as interleukin (IL)1RA, IL-1β, IL-6, tumor necrosis factor α (TNF- α), monocyte chemotactic protein-1 have been reported in the plasma of pregnant women with pPROM [38]. In a previous investigation of ours, cytokine levels in the vaginal fluid were higher among pregnant women who delivered preterm with pPROM and PTL compared to women with term deliveries [4]. We suggest that further studies are need to investigate the link between methylation of VTRNA2-1 promoter and cytokine in maternal blood.

VTRNA2-1 is a major candidate for an environmentally responsive epiallele and its expression is regulated to epigenetic silencing by promoter methylation [39], which could be modulated via either stress, chemotherapy or TGF-β cytokine activity [25, 34]. Levels of VTRNA2-1 methylation are similar among normal mature B cells, T cells and granulocytes [25]. In addition, VTRNA2-1 is reported to have DM sites in various regions and is associated with several diseases [25, 26]. Although few studies have investigated the relationship between VTRNA2-1 and PTB, the elevated methylation is associated with poor outcomes in patients with acute myeloid leukaemia and small cell lung cancer [25, 26]. Moreover, inhibition of VTRNA2 increases Bax protein expression and apoptotic cell death in cervical cancer cells [40].

One study reported that the methylation level of VTRNA2-1 was associated with the season of conception and maternal nutrition in rural Gambia [34]. Specifically, the authors reported that women had differing nutrition in the dry and rainy seasons during pre-conception. However, although our VTRNA2-1 methylation data were associated with season of delivery, it is difficult to explain the relationship between PTB and methylation changes by season. Another studies reported that alterations in DNA methylation result from inflammatory processes, such as circulating levels of C-reactive protein (CRP) or other inflammatory proteins [41, 42]. In this study, level of VTRNA2-1 methylation is correlated with circulating level of CRP in PTB and CRP level is correlated with WBC count (data now shown). Therefore, our study suggests the possible that elevated VTRNA2-1 is susceptible to PTB by inducing infection-inflammation pathways.

Our results showed that expression of VTRNA2-1 was negatively correlated with methylation levels, and that elevated methylation of the VTRNA2-1 promoter was associated with PTB. VTRNA2-1 is a putative tumour suppressor and modulator of innate immunity [26]. VTRNA2-1 expression is suppressed in clinical tumour samples compared to normal tissues; more importantly, low expression of VTRNA2-1 is associated with poor survival. In addition, inhibition of VTRNA2-1 leads to activation of the cellular antiviral response pathways involving protein kinase-R (PKR) [43–45]. PKR activation can be induced by various stressful stimuli, such as cytotoxic cytokines, growth factor deprivation and DNA damage [45]. As mentioned above, proinflammatory cytokines are associated with increased risk of PTB. Thus, we propose the possibility that elevated methylation of VTRNA2-1 could cause PTB due to PKR activation in response to increased cytokine.

In our results, the methylation levels of VTRNA2-1 were divided into either a 30–60 % group or 0–13 % group. The pyrosequencing result from our cohort showed hypomethylation at the VTRNA2-1 promoter in 28 (34.1 %) samples. To assess the occurrence of this hypomethylation in other populations, we obtained an independent genomic DNA methylation dataset (GSE168406) generated from 136 peripheral blood samples of Indian pregnant women who delivered at term (n = 68) or preterm (n = 68). The methylation status of 6 probes at VTRNA2-1 promoter in this dataset was then analysed (supplementary methods) and methylation levels of cg04481923, cg18678645, cg00124993, and cg26896946 were significantly elevated in PTB compared to TB (data not shown). In addition, hypomethylation occurred at 25 %~48.5 % of blood samples (Table S5), on an average of 34.2 % of the population, which was consistent with our statistic of 34.1 % in the Korean population. These results indicate that elevation of VTRNA2-1 promoter is associated with PTB and the hypomethylation of VTRNA2-1 promoter is common in both Korean and Indian populations.

We acknowledge several limitations of our study, including the paucity of clinical and demographic data, for example on smoking status, alcohol consumption, psychiatric disorders and the use of other drugs or medications. We were only able to demonstrate a correlation between methylation and expression of VTRNA2-1 in a small subset of CpG sites; moreover, the sample size was small due to the limited availability of RNA from maternal blood samples. Finally, when we analysed the VTRNA2-1 methylation level using a simple linear regression model that included the cell composition percentages as covariates, the VTRNA2-1 methylation level was not affected by cell composition (*p* > 0.05). However, we did not analyse DNA methylation status according to cellular heterogeneity, because we did not sort the samples by blood cell type at the time of collection.

## Conclusions

This study suggests that VTRNA2-1 methylation, identified through genome-wide DNA methylation analysis and verification by pyrosequencing in blood cells, may be associated with PTB. Our findings suggest that elevated methylation of VTRNA2-1 promoter appear more frequently in pPROM than in PTL, and may be associated with increased risk of PTB. Thus, the evaluation of genes containing these differentially methylated sites may be useful to identify biological pathways involved in PTB, thereby facilitating the identification of clinically informative biomarkers for the prediction of PTB.

## Methods

### Study population

We conducted a case-control study of 10 pregnant women with term (*n* = 5) and preterm deliveries (*n* = 5) at Ewha Womans University Mokdong Hospital (Seoul, Korea) to screen changes in methylation level. Maternal peripheral blood samples from participants were collected at the time of delivery, and the birth outcome was followed (Table S2). DNA methylation was measured using the Illumina Human Methylation 450 BeadChip. To validate the DM levels, 82 blood samples from women with term (*n* = 39) and preterm (*n* = 43) births were examined. All participants gave informed consent, and the study was approved by the Institutional Review Board of Ewha Womans University Mokdong Hospital (Certificate No. EUMC 2014-06-010-003, Samsung Medical Center (SMC 2014-06-094-003), Konkuk University Medical Center (KUH1040034), and Seoul St. Mary’s Hospital (KC14TIMI0591). Informed consent was obtained from all subjects involved in the study. Women with multiple births, major birth defects, haemolysis, elevated liver enzymes, a low platelet count (HELLP) syndrome, preeclampsia and gestational diabetes mellitus were excluded. Additionally, pregnant women with inflammatory and metabolic diseases or hormonal problems also were excluded. Gestational age was determined using the first day of the last menstrual period and ultrasound examination.

### DNA preparation and genome-wide DNA methylation analysis

Maternal blood was collected in EDTA tubes, and the plasma was separated and stored at -80 °C. Genomic DNA was extracted from blood samples using the QIAGEN Mini Kit (QIAGEN, Valencia, CA, USA) following the manufacturer’s protocol. The quality of the extracted DNA was evaluated using agarose gel electrophoresis. To analyse DNA methylation, ~ 700 ng genomic DNA was bisulphite-converted using the Zymo EZ DNA Methylation Kit (Zymo Research, Irvine, CA, USA), amplified, fragmented, and hybridised to the Illumina Infinium HumanMethylation450 BeadChip (Illumina, San Diego, CA, USA) following the manufacturer’s protocol. After washing, the BeadChips were scanned with the HiScan SQ System (Illumina). Scanned images were processed to determine the signal intensity and β-values were calculated using Genome Studio software (Illumina). The β-value, as defined below, was used to measure methylation levels on a scale from 0 to 1:

$$\mathrm\beta=\frac{Max\mathit\;{\mathit(Signal\mathit\;B\mathit.\mathit0\mathit)}}{Max\mathit\;{\mathit(Signal\mathit\;A\mathit.\mathit0\mathit)}\mathit+Max\mathit\;{\mathit(Signal\mathit\;B\mathit.\mathit0\mathit)}\mathit+\mathit{100}}$$

Max (Signal A,0) indicates the signal intensity of the unmethylated allele, and Max (Signal B,0) indicates the signal intensity of the methylated allele. A constant bias of 100 was added to regularise the β-value. The β-values were calculated; normalisation, filtration, and statistical analyses were performed using GeneSpring ver. 7.3 (Agilent Technologies, Santa Clara, CA, USA). The normalised β-value of all CpG sites in the two groups (term vs. preterm) were statistically evaluated using Welch’s t-test (*p* < 0.05). We accounted for multiple testing by controlling for the false discovery rate (FDR). The FDR was controlled using the Benjamini–Hochberg correction (*q* < 0.05).

### DM analysis by pyrosequencing

DM levels measured by the genome-wide methylation array were validated in maternal term (*n* = 39) and preterm (*n* = 43) blood by pyrosequencing. The cg04481923 site was amplified using a primer set designed using PSQ Assay Design software (Biotage AB, Uppsala, Sweden) (Table S3). Genomic DNA was bisulphite-converted according to the manufacturer’s instructions with an EZ DNA Methylation Kit (ZYMO Research, Irvine, CA, USA). An EpiTect PCR Control DNA Set (Qiagen) was used as a methylated/unmethylated control. The percentage of methylated cells in each region was quantified using the PyroMark ID pyrosequencer (Qiagen) and Pyro Q-CpG Software (Figure S2). The software incorporates controls to check for completed bisulphite conversions, and provides an adequate signal over background noise. All samples were run in duplicate and average values were calculated. The details of the pyrosequencing methodology have previously been reported [46, 47].

### RNA isolation and quantitative real-time polymerase chain reaction

Total RNA from maternal blood (*n* = 40) was extracted using the Easy-BLUE™ Kit (iNtRON Biotechnology, Sungnam, Korea) according to the manufacturer’s instructions. RNA was reverse transcribed using 1 µg total RNA in a 25 µL reaction mixture containing 1 µL 10 pM oligonucleotide primer, 5 µL 10× reverse transcription buffer, 5 µL 2.5 mM dNTPs, 1 µL 20 U RNase inhibitor, and 1 µL 200 U Moloney murine leukaemia virus reverse transcriptase (M-MLV RT) (Promega, Madison, WI, USA) for 60 min at 42 °C. Real-time quantitative-polymerase chain reaction (qPCR) was performed using synthesised cDNA as a template, gene-specific primers (VTRNA2-1), and Power SYBR Green PCR Master Mix (Applied Biosystems, Foster City, California, USA). The reactions (including the no-template controls) were run in duplicate on the ABI PRISM 7000 sequence detection system (Applied BioSystems) using glyceraldehyde-3-phosphate dehydrogenase (GAPDH) as an internal reference for normalisation of target gene mRNA expression. The PCR conditions were as follows: denaturation at 95 °C for 30 s, 40 cycles of denaturation at 95 °C for 15 s, and annealing/extension at 60 °C for 1 min. We tested primer specificity by RT-PCR and confirmed it using melting (dissociation) curve analysis. Comparative quantification of each target gene was performed based on the cycle threshold (CT), which was normalised against the CT of GAPDH using the ΔΔCT method. Data are presented as the fold change between groups as the mean ± standard error of the mean (SEM). The primer sets and melting temperature (Tm) for qPCR are described in Table S4.

### Statistical analysis

The basic characteristics of the study groups were compared using Student’s *t*-test for continuous variables and the chi-square test for categorical variables. After pyrosequencing, the DNA methylation levels between the two groups were compared using the Mann–Whitney U-test. DNA methylation levels of VTRNA2-1 were analyzed in two separate groups, a group with hypomethylation (< 13 %) and a group with elevated levels of methylation (30–60 %) by the level of methylation. We used the Spearman coefficient to analyze the correlation between VTRNA2-1 methylation levels and maternal clinical characteristics. To explore the association between VTRNA2-1 methylation level and PTB, multiple logistic regression was conducted, controlling for maternal age, parity, season, and white blood cell (WBC) count. In addition, the clinical characteristics of the VTRNA2-1 hypo- and elevated methylation groups were analysed using Student’s *t*-test and the chi-square test. All analyses were two-tailed, and a *p*-value < 0.05 was considered statistically significant. All statistical analyses were performed using SPSS software ver. 21.0 (IBM, Armonk, NY, USA).

## Supplementary Information

**Additional file 1: Table S1. **Differentially methylated sites between term and preterm samples (*n* = 1,581).

**Additional file 2: Table S2. **The demographics from the pilot cohort (*n *= 10).

**Additional file 3: Table S3. **Primer set for pyrosequencing designed by the PSQ Assay Design software.

**Additional file 4: Table S4. **Primers sets for Quantitative real-time PCR.

**Additional file 5: Table S5. **Frequency of hypomethylation of the VTRNA2-1 promoter in Indian populations (*n* = 136).

**Additional file 6: Figure S1. **Comparison of seven differential CpG sites on VTRNA2-1 between women with term and preterm births in initial samples (*n* = 10).

**Additional file 7: Figure S2. **Schematic representation of the human VTRNA2-1 gene. (a) Identified CpG site of VTRNA2-1 (b) PCR products (c) Pyrogram.

## Data Availability

The datasets supporting the conclusions of this article are included in the article and its Additional files. The datasets generated and/or analysed during the current study are available in the GEO repository, https://www.ncbi.nlm.nih.gov/geo/ (accession numbers: GSE178609).

## Abbreviations

VTRNA2-1: Vault RNA 2-1
PTB: Preterm birth
BMI: Body mass index
PKR: Protein kinase-R
CRP: C-reactive protein
GAPDH: Glyceraldehyde-3-phosphate dehydrogenase
CT: Cycle threshold
SEM: Standard error of the mean
pPROM: Preterm premature rupture of membrane
PTL: Preterm labor
