# Supplementary material for: Elevated methylation of the vault RNA2-1 promoter in maternal blood is associated with preterm birth
Source: BMC Genomics. 2021 Jul 10;22:528. doi: 10.1186/s12864-021-07865-y (PMC8272312; doi:10.1186/s12864-021-07865-y)
Supplement: Supplementary file 2 — Additional file 2: Table S2. The demographics from the pilot cohort (n = 10). [file 12864_2021_7865_MOESM2_ESM.docx]

Table S2. The demographics from the pilot cohort (n=10).

|  | Term (≥ 37, *n* = 39) | Preterm (< 37, *n* = 43) | *p*-value |
| --- | --- | --- | --- |
|  | Mean ± SD | Mean ± SD |  |
| Maternal age | 34.8 | 34.0 | 0.79 |
| BMI | 28.1 | 23.5 | <0.01^a^ |
| Gravidity |  |  |  |
| 0 | 2 | 1 |  |
| 1 | 3 | 4 |  |
| Parity |  |  |  |
| Nulliparous | 2 | 1 |  |
| Multiparous | 3 | 4 |  |
| Delivery season |  |  |  |
| Spring, *n* (%) | 2 | 0 |  |
| Summer, *n* (%) | 0 | 1 |  |
| Autumn, *n* (%) | 3 | 2 |  |
| Winter, *n* (%) | 0 | 2 |  |
| Mode of delivery |  |  |  |
| Vaginal, *n* (%) | 5 | 5 |  |
| Gestational age | 39.0 | 31.7 | <0.001^a^ |
| Birth weight | 2055 | 3230 | <0.001^a^ |

BMI, body mass index. ^a^ Student’s t-test
