# Supplementary material for: Elevated methylation of the vault RNA2-1 promoter in maternal blood is associated with preterm birth
Source: BMC Genomics. 2021 Jul 10;22:528. doi: 10.1186/s12864-021-07865-y (PMC8272312; doi:10.1186/s12864-021-07865-y)
Supplement: Supplementary file 3 — Additional file 3: Table S3. Primer set for pyrosequencing designed by the PSQ Assay Design software. [file 12864_2021_7865_MOESM3_ESM.docx]

Table S3. Primer set for pyrosequencing designed by the PSQ Assay Design software.

| Target ID |  | F1 | R1 | S1 | TM  (F) | TM  ('R) |
| --- | --- | --- | --- | --- | --- | --- |
| cg04481923 | Pos1 | GTAAAGTTAAAAGGGATAAAAAA | AACTTTCTATCTATCCATCTCTAT | ATAAAAGGGTTAGTAAGTAT | 60.1 | 58.5 |
