# Supplementary material for: Elevated methylation of the vault RNA2-1 promoter in maternal blood is associated with preterm birth
Source: BMC Genomics. 2021 Jul 10;22:528. doi: 10.1186/s12864-021-07865-y (PMC8272312; doi:10.1186/s12864-021-07865-y)
Supplement: Supplementary file 4 — Additional file 4: Table S4. Primers sets for Quantitative real-time PCR. [file 12864_2021_7865_MOESM4_ESM.docx]

Table S4. Primers sets for Quantitative real-time PCR.

| Target ID | F1 | R1 | TM  (F) | TM  ('R) |
| --- | --- | --- | --- | --- |
| VTRNA2-1 | CACACCTTCAAAGTGACACCA | TCCTCACGGACTTTCTA | 67.1 | 65.8 |
