# Supplementary material for: Elevated methylation of the vault RNA2-1 promoter in maternal blood is associated with preterm birth
Source: BMC Genomics. 2021 Jul 10;22:528. doi: 10.1186/s12864-021-07865-y (PMC8272312; doi:10.1186/s12864-021-07865-y)
Supplement: Supplementary file 5 — Additional file 5: Table S5. Frequency of hypomethylation of the VTRNA2-1 promoter in Indian populations (n = 136). [file 12864_2021_7865_MOESM5_ESM.docx]

Table S5. Frequency of hypomethylation of the VTRNA2-1 promoter in Indian populations

| Target ID | Term (≥ 37, *n* = 68) | Preterm (< 37, *n* = 68) | Total |
| --- | --- | --- | --- |
| Cg04481923 | 30 (44.1%) | 22 (32.4%) | 52 (38.2%) |
| Cg18678645 | 35 (51.5%) | 31 (45.6%) | 66 (48.5%) |
| Cg25340688 | 24 (35.3%) | 12 (17.6%) | 36 (26.5%) |
| Cg00124993 | 29 (42.6%) | 21 (30.9%) | 50 (36.8%) |
| Cg06536614 | 22 (32.4%) | 12 (17.6%) | 34 (25.0%) |
| Cg26896946 | 25 (36.8%) | 16 (23.5%) | 41 (30.1%) |

Dara were presented as the number of samples (%). We obtained a DNA methylation dataset (GSE168406) in the GEO database, which was generated from peripheral blood DNAs of 136 Indian mothers using Infinium EPIC Human DNA methylation BeadChip v1.2.8. We first normalized the beta-values using a quantile normalization method (PMID: 12538238) and then identified hypomethylated CpG sites as the ones with beta-values < 0.2, as previously described (PMID: 31035926). In the DNA methylome data, beta-values were available for six (cg04481923, cg18678645, cg25340688, cg00124993, cg06536614, and cg26896946) of the seven CpG sites in VTRNA2-1 analyzed in this study. For each of the six CpG sites, hypomethylation was observed in 25%~48.5% of the 136 samples, with an average of 34.2%.
