# Supplementary material for: Elevated methylation of the vault RNA2-1 promoter in maternal blood is associated with preterm birth
Source: BMC Genomics. 2021 Jul 10;22:528. doi: 10.1186/s12864-021-07865-y (PMC8272312; doi:10.1186/s12864-021-07865-y)
Supplement: Supplementary file 6 — Additional file 6: Figure S1. Comparison of seven differential CpG sites on VTRNA2-1 between women with term and preterm births in initial samples (n = 10). [file 12864_2021_7865_MOESM6_ESM.docx]

Figure S1. Comparison of seven differential CpG sites on VTRNA2-1

between women with term and preterm births in initial samples (n=10).
