# Supplementary figures and images for: Elevated methylation of the vault RNA2-1 promoter in maternal blood is associated with preterm birth
Source: BMC Genomics. 2021 Jul 10;22:528. doi: 10.1186/s12864-021-07865-y (PMC8272312; doi:10.1186/s12864-021-07865-y)

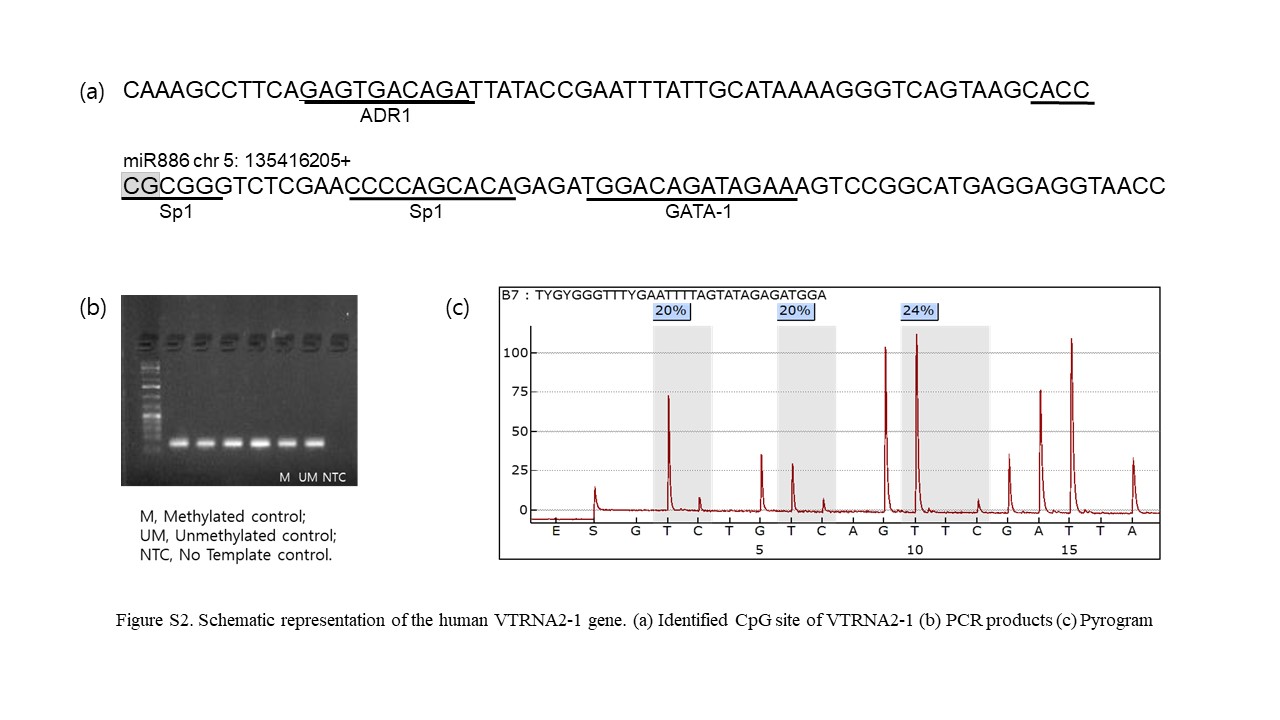

Supplement: Supplementary file 7 — Additional file 7: Figure S2. Schematic representation of the human VTRNA2-1 gene. (a) Identified CpG site of VTRNA2-1 (b) PCR products (c) Pyrogram. [file 12864_2021_7865_MOESM7_ESM.docx]
